# Adherence to Gluten-Free Diet in Children with Celiac Disease

**DOI:** 10.3390/nu10101424

**Published:** 2018-10-04

**Authors:** Grażyna Czaja-Bulsa, Michał Bulsa

**Affiliations:** 1 Department of Pediatric Diseases and Pediatric Nursing, Clinic of Pediatrics, Gastrology and Childrens Rheumatology, Pomeranian Medical University, 71-454 Szczecin, Poland; 2 Department of Gynecological Surgery and Gynecological Oncology of Adults and Adolescents, Pomeranian Medical University, 71-454 Szczecin, Poland; michal.bulsa@gmail.com

**Keywords:** gluten-free diet, celiac disease, children, teenagers

## Abstract

Celiac disease (CD) can only be treated by rigorous life-long gluten-free diet (GFD). The study included 102 mothers and their CD children treated with GFD for at least two years. Frequency and cause of diet failure in children treated at present (54 children) and 10 years ago (48 children) were compared. Dietary adherence was evaluated serologically (tTG), while diet management difficulties were examined by means of a questionnaire. The study shows that one-third of patients fail to follow GFD, more often 10 years ago than now (40% vs. 26%; *p* < 0.05), mainly children aged 13–18 (54% vs. 40% now; *p* < 0.05). Younger children (up to 12) are less likely to abandon the diet (27% vs. 8%; *p* < 0.05). In this age group non-intentional diet failure prevails, while teenagers interrupt their diet intentionally (45% vs. 33%; *p* = ns (small population of children in this groups)). Currently, the most common causes of teenage diet failure are the absence of symptoms after consuming a small amount of gluten and, even more often, troublesome diet administration. Previously, the absence of peer acceptance prevailed. With this study we found that: 1. In West Pomerania, every fourth CD child does not follow GFD. 2. For years, teenagers have failed to follow GFD due to the absence of symptoms after consuming small amounts of gluten. 3. The incidence of non-intentional failure to follow GFD has significantly decreased over years, which indicates better dietary care.

## 1. Background

Celiac disease (CD) is a genetically conditioned, immunologically mediated chronic intestinal disease, in which in genetically predisposed people the consumption of gluten leads to the disappearance of intestinal flora [1]. This results in reduced nutrient absorption. The disease frequency is steadily increasing—in Europe and America it affects 1% of people. The only effective way of its treatment is life-long strict adherence to a gluten-free diet (GFD) [1]. The gluten-free diet consists in the complete elimination of products derived from wheat, rye, and barley and products processed from these cereals. They are replaced by naturally gluten-free products (maize, rice, oats, buckwheat, lamb, meat, fish, vegetables, fruit) or by products from which gluten has been removed. Adherence to GFD leads to the regeneration of intestinal villi after a period of 6 to 24 months. The life-long GFD secures a child's proper development and protects them from ailments such as abdominal pain, flatulence, loose stools, constipation, iron deficiency anemia, low stature, and osteoporosis. In adults with CD, it also reduces the risk of cardiovascular diseases and intestinal tumors [1,2].

The most reliable method to control GFD adherence are serological tests and small intestine biopsy [1]. It is recommended to determine serum tissue transglutaminase concentration in IgA class (IgA-tTG), while in patients with selective IgA deficiency—in IgG class (IgG-tTG) [1]. The determination of tTG concentration in patients treated with GFD is commonly referred to as the ‘lie test’ because it allows to identify people who do not follow the diet. Being an invasive test, endoscopic small intestine biopsy is not recommended to confirm non-adherence to GFD.

Some researchers suggest using nutritional history to assess the GFD adherence. However, it does not allow to identify patients who non-intentionally fail to adhere to GFD, therefore its importance for monitoring procedures is low. Leffler et al. proposed a seven-point questionnaire to identify patients who did not adhere to GFD (Celiac Dietary Adherence Test, CDAT). The test assesses their knowledge of the disease, which, according to Leffler, is highly correlated with serological test results [3]. In 2017, a new method was proposed to confirm the consumption or nibbling of gluten by people with CD. The method consists in determination of gluten immunogenic proteins in urine [4]. This is a very attractive proposal as the test is non-invasive.

Adherence to GFD is troublesome [5]. The patients or their guardians are required to have extensive knowledge about CD and the GFD requirements. The changes in eating habits affect not only the patient, but often their whole family. According to Meyer et al., in order to better manage CD the daily routines of young patients should be carefully examined to help them strictly comply with GFD [6]. The GFD preparation time is much longer than that of a diet containing gluten [7]. Also, it is much more expensive than traditional nutrition, which limits its availability to many patients. Fortunately, in some countries the costs of GFD are partially refunded [8,9]. It was noted that in such countries the patients who receive products free of charge follow the GFD requirements more often [7,10]. For the low-income adult patients the high cost of GFD products is a barrier to diet adherence [11]. On the other hand, the studies of Humayun et al. and Leffler et al. did not confirm the significant influence of the GFD price on the frequency of the GFD adherence, except for those CD adult patients who openly declared that the high price of products made it difficult for them to follow the diet [3,7].

In the opinion of some authors, the following factors contribute to the better adherence to GFD: good knowledge of the disease and its treatment, higher education level, better social situation of the family, female sex, young age, high self-esteem, good grades at school, good availability and labelling of products, good contact with a doctor and a dietitian, and finally, membership in the Coeliac Society [5,7,11,12,13]. The Coeliac Society members understand the disease better and know how to prepare gluten-free meals. They also receive stronger day-to-day support (meetings, help in obtaining GFD products, partially reimbursed youth camps) [7,10,14].

The factors responsible for not adhering to GFD are: poor taste of gluten-free products, their high price and low availability (especially during travel and social meetings), the patient's adolescence, the absence of immediate symptoms after consuming small amounts of gluten, and low awareness of the disease [5,11,13]. 

Better awareness of the factors that have significant impact on the GFD adherence can improve the supervision of CD patients.

The aim of this study was to determine the incidence and causes of non-adherence to GFD by children with CD treated now and 10 years ago.

The first analysis of the causes of failure to adhere to GFD was carried out in 2006–2007 with a view to improving the effectiveness of CD treatment, as it had been observed that many patients discontinued GDF after years of adherence. In recent years, GFD has become popular in Poland and therefore the conclusions concerning the patients’ failure to adhere to GFD 10 years ago are no longer relevant. Unfortunately, it has been observed that teenagers still tend to discontinue their GFD. Therefore, in the 2016–2017 study, the questions about the reason for failure to follow GFD were again included in the CD patients’ medical history.

## 2. Materials and Methods

The study covered 102 children (64 girls) with coeliac disease (CD) treated with GFD. All patients were diagnosed in accordance with the applicable criteria: elevated antibodies anti–endomysium (EMA) and anti-tissue transglutaminase (tTG) as well as duodenal atrophy of intestinal mucosa [1]. There were no patients with selective IgA deficiency in the study group, therefore antibodies were determined only in the IgA class. At the time of their CD diagnosis the mean IgA-EMA concentration was 1:120 (range: 1:170–1:10) and IgA-tTG was 171 RU/mL (range: 297 RU/mL–38 RU/mL). During the first two years after the diagnosis, serological indicators (EMA and tTG) normalized in all patients, in most of them already in the first year of treatment. It is believed that in patients treated with GFD, a two-year period is sufficient to fully normalize the antibodies concentration and the intestinal mucosa [15]. 

The patients were recruited during their GFD treatment, two years or more after the diagnosis, during control tests performed routinely twice a year. The mean time of the GFD treatment before their inclusion in the study was 104 months (28 months–208 months). 

During the medical interview, patients made a declaration concerning their dietary adherence. If they reported that they did not follow the diet, they were asked to give reasons. Five probable causes were specified: poor taste of the diet, its high price, troublesome dietary regime (understood as problems with the purchase of gluten-free diet, its labelling, the inability to eat outside the home), difficulties in relationships with peers (due to being rejected by the peer group) and the absence of symptoms after consuming gluten products. Each patient was asked to choose which of the reasons was the most relevant to them, i.e., which led to deliberately abandoning of GFD. In the case of the youngest children (under seven years of age), dietary declarations were made by their guardians (in this study all of them were mothers). Older children with CD and their guardians jointly chose the most important reason for their failure to follow GFD.

The tests performed during each control visit included serum assays of tissue transglutaminase (tTG) in the IgA class. The presence of tTG antibodies in serum confirmed that the CD patient had consumed gluten. Patients who claimed to adhere to GFD and in whose blood tTG antibodies were found were considered as those who non-intentionally failed to adhere to GFD. In 33 children who did not follow GFD, the average IgA-tTG concentration was 126 RU/mL (264 RU/mL–22 RU/mL). In children adhering to GFD the IgA-tTG concentration was below 20 RU/mL. 

Patients were divided into two groups: children (0–12 years old) and teenagers (13–18 years old) (Table 1). The frequency and reason for non-adherence to GFD was compared between children treated now (54 children) and 10 years before (48 children).

EMA was determined by indirect immunofluorescence test (standard < 1:10), while IgA-tTG was assayed by ELISA (standard < 20 RU/mL). The obtained results were subjected to statistical analysis. The Student’s *t*-test was used for independent trials.

The survey did not require the permission of the PUM (Pomeranian Medical University) Bioethical Committee. The relevant opinion was obtained from the Chairman of the Committee in 2006. All subjects had given their informed consent for inclusion before they were included in the study.

## 3. Results

**Figure 1:**
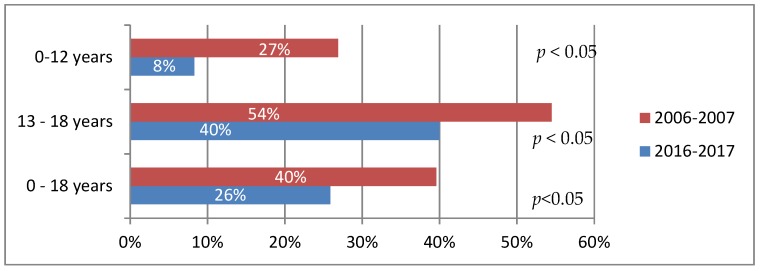
Proportion of children failing to adhere to gluten-free diet (GFD) by age and time of study.

**Figure 2:**
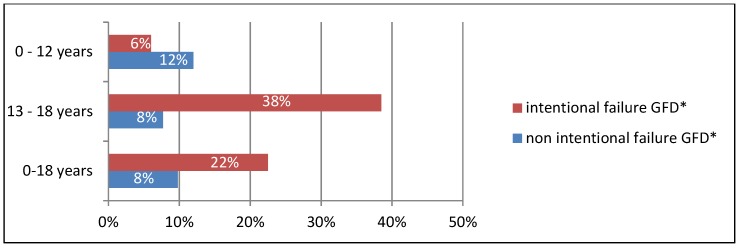
Incidence to intentional and non-intentional failure to adhere to gluten-free diet (GFD). * GFD—gluten-free diet.

**Figure 3:**
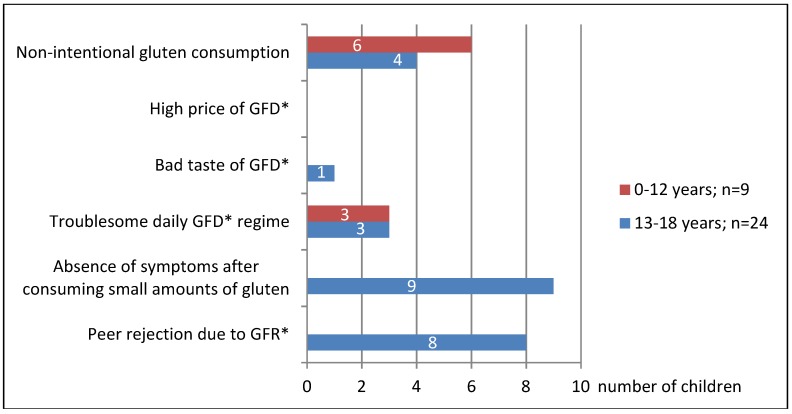
Reasons for non-adherence to gluten-free diet (GFD) by age. * GFD—gluten free diet.

**Figure 4:**
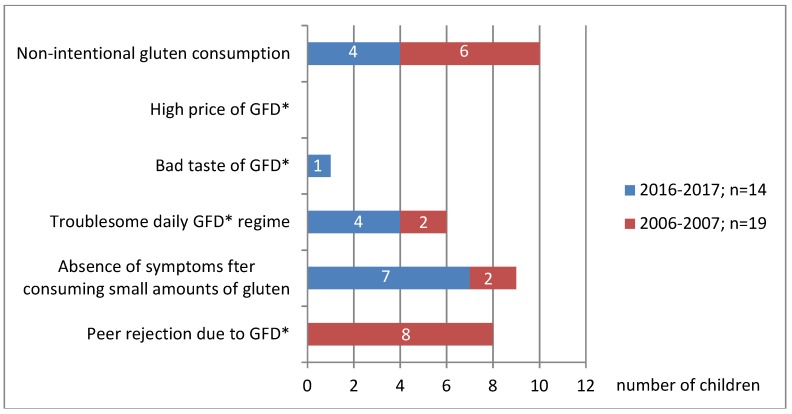
Reasons for GFD non-adherence by time of study. * GFD—gluten-free diet.

It has been shown that children with CD are now more likely to adhere to GFD than before. In 2016–2017, 26% of children did not adhere to GFD, whereas 10 years earlier, in 2006–2007, as many as 40% failed to do so (*p* < 0.05) (Figure 1). Those were mainly children aged 13–18 (40%) by age (cumulative assessment of 2006–2007 and 2016–2017 studies) now and 54% a decade ago; *p* < 0.05). Younger children (up to 12 years) were less likely to miss the diet (8% at present and 27% a decade ago; *p* < 0.05). At that age, the incidence of non-intentional GFD failure was larger: 4% at present (1 of 24 children) and 19% previously (5 of 26 children) (Figure 2). Teenagers often intentionally interrupt their diet: 33% now (10 of 30 children) and 45% a decade ago (10 of 22 children) (Figure 2). Currently, the most common reason for the teenage diet failure is the lack of symptoms after consuming a small amount of gluten products, while the inconvenience of GFD regime is less common (Figure 3 and Figure 4). In 2006–2007, the main reason for GFD non-adherence was the difficulty in peer relations (rejection by peer groups), while the absence of symptoms after occasional gluten intake or troublesome dietary regime were less common (Figure 3 and Figure 4).

## 4. Discussion

The analysis shows that currently every fourth child with CD does not adhere to GFD (Figure 1). Ten years ago, in 2006–2007, the failure to adhere to GFD was more common, and was reported by as many as 40% of the respondents. Studies in other countries have shown that between 30% and 75% of patients fail to follow GFD [7,11,14,16]. In Polish multi-center studies conducted in 2010—2013 and covering 277 children with CD, it was found that GFD was not observed by 25% of patients, of whom 19% had abandoned the diet intentionally [17].

Currently, teenagers are five times more likely not to adhere to GFD than younger children (40% vs. 8%). In 2006–2007, teenagers consumed gluten only twice as often as younger children (54% vs. 27%). Similar observations were made by other researchers [5,12,18]. What has clearly decreased in recent years is the incidence of dietary failure among younger children, who most often fail to follow GFD unintentionally (Figure 2). In this group, the intentional failure to adhere to the diet was primarily a consequence of their parents' inability to provide them with a sufficient amount of gluten-free products. In those years, the access to such products was much more difficult than today. Currently, CD families are better prepared to adopt GFD. The improved ability of parents and children to identify gluten-free products is a consequence of better dietary support, greater involvement of the Coeliac Society (regular meetings, publications), improved labelling of gluten-free products and a more convenient way to obtain them (online shopping).

Both 10 years ago and now, the majority of CD teenagers intentionally fail to adhere to GFD (Figure 2), which has also been found out by other authors [5]. Currently, the main reason for their failure to adhere to GFD is that they believe that the systematic low intake of gluten does not affect their health, as it does not cause any intestinal symptoms which they experienced before (Figure 3 and Figure 4). Unfortunately, non-adherence to GFD at this age does not correlate well with the presence of the CD symptoms.

In younger children (<3 years), the consumption of even the smallest amounts of gluten usually result in intestinal symptoms, most commonly abdominal pain or abnormal stools. Teenagers, seeing that such a co-incidence no longer occurs, start to believe that they can consume gluten in small amounts. There are patients who are able to precisely define the amount of gluten that cause the symptoms. Therefore, they try to consume smaller amounts, which in their opinion does not harm their health. In this group of patients, dietary errors are caused by insufficient knowledge of CD and its treatment. They need to be informed about the CD again. It seems that such information should be provided to every juvenile patient once they enter adolescence. It should be particularly pointed out that, at this age, eating small amounts of gluten may not cause such intestinal discomfort as previously, but it still remains detrimental to their health. We try to persuade these patients to adhere strictly to GFD by showing them that their growth rate has been slowed down, anemia induced by iron deficiency has occurred, bone density has decreased (in densitometric evaluation), and antibodies indicating CD activity have appeared in serum. In some cases, we repeat endoscopic tests of fragments from the duodenum in order to show that intestinal villi atrophy has reoccurred in their duodenum. We explain that these are the complications due to the young patient's failure to adhere to GFD and that they have appeared independently from the absence of intestinal discomfort. Systematic monitoring of teenagers' dietary adherence is absolutely essential, even if they have had a history of strict GFD adherence. Other authors also believe that the lack of immediate symptoms after consumption of small amounts of gluten is an important factor in teenagers' failure to adhere to GFD [5].

Ten years earlier, in 2006–2007, the main cause of non-adherence to GFD by teenagers was peer rejection due to their different dietary regime. These children were stigmatized by other children and isolated from the group. In order not to be socially excluded, teenagers with CD intentionally consumed gluten products while in social situations. None of these children changed their behavior throughout the treatment period (up to 18 years), despite having been informed about the adverse effects of such behavior. Similar teenagers’ attitudes have also been described by other authors [5,6,16,19]. Swedish studies have found that girls are more likely than boys to follow GFD [20]. 

In our 2016–2017 study, none of the children reported the above as a reason for non-adherence to GFD. This is a consequence of a change in social customs. Today, GFD has become popular in Poland and it is used by many people who do not suffer from CD. Similar trends have also been observed in other countries where the frequency of GFD consumption far exceeds the number of CD patients [19,21,22].

Younger children have never reported this problem. In kindergartens and schools, they consumed gluten-free products and that fact did not have any negative impact on their peer relations.

At present, just like 10 years ago, the second most frequent reason for non-adherence to GFD is its troublesome administration, as reported by both younger children and adolescents (Figure 3 and Figure 4). By the troublesome use of GFD we mean a number of difficulties encountered by a patient in everyday life. These are: problems with buying GFD and with label reading, as well as the inability to eat out, which makes travelling and social gatherings more difficult. In the years 2006–2007 in Poland the purchase of gluten-free products was possible only in specialist shops located in large cities. That significantly hindered access to gluten-free food for the residents of small towns and villages. It forced people to prepare gluten-free products (bread, cakes, pasta) on their own at home. There were no possibilities to order gluten-free meals outside one’s place of residence. The children went on trips with their own bread, which significantly limited their ability to travel. The only form of organized tourism were camps organized by the Coeliac Society, but they were not widely available. The situation is now much better. Most families buy gluten-free products online, and in many towns and cities there are gluten-free food stands in supermarkets. There is a wide offer of children and youth camps serving gluten-free menu that are organized not only by the Coeliac Society. However, the possibility of eating out is still very limited, even though the situation has improved considerably in recent years. Currently, GFD has become ‘trendy’ in Poland, it is chosen by many people, not only by the CD patients. In many restaurants you can order gluten-free menu. Unfortunately, most of such places are usually located in large towns, which still hampers the CD patients’ mobility. Similar difficulties are also highlighted by other authors from other countries [5,7,13,23]. In Wolf et al. studies, difficulties with eating outside the home were more frequently reported by teenagers than by adults (87% vs. 74%) [24]. In our study on teenagers, that was the main factor indicating the troublesome character of the diet. Parents of younger children (<12 years old) reported difficulty in reading labels on gluten-free products, their high price, and difficult access. 

When buying gluten-free products, patients can benefit from a mobile application containing an inventory of gluten-free products available in a given country [25]. Such an application has been available in Poland since spring 2017. It is free of charge for patients with CD belonging to the Coeliac Society. In Canada, as many as a quarter of patients reported that it would be very helpful to include a list of 100 most frequently purchased gluten-free products in their mobile applications. Silvester et al. have shown that in the USA only 25% of adults with CD are able to recognize the majority of gluten products from the list (14 out of 17 products) [14]. 

In our studies none of the CD patients gave a high GFD price as the main reason for not following it. That was another, but not the most important, reason. During the 2006–2007 study, all Polish children with CD received a cash allowance to buy GFD. Unfortunately, today the allowance is not available to the majority of children. Many authors point out that the high price of GFD is an important reason for not adhering to GFD by many patients [11,13,26]. It is also an essential factor affecting the quality of life of adult patients with CD [27].

None of the examined groups of children indicated the illegible labelling of the gluten-free products as the most important reason for not adhering to GFD, which has also been reported by other researchers [7,8,13]. 

Strict adherence to GFD is most common among patients with greater knowledge of their disease [24]. They are ‘extremely vigilant’ in selecting the right products. They eat meals only at home, as they are afraid to eat out. They are constantly using the Internet and mobile applications to help them buy GFD products. They are more likely to experience anxiety and fatigue. Their quality of life (QoL) is significantly lower than that of patients who are ‘less vigilant’ in the choice of GFD [24]. This applies to teenagers as much as to adults, more often to women [27]. This is a negative consequence of strict adherence to the diet and points to the need to provide psychological and emotional support for the patients with CD. The opposite results were obtained by other researchers in their studies on adults which revealed better quality of life reported by patients strictly adhering to GFD [5,28]. The discrepancies between the two findings have been explained by Nachman et al. who showed that in the first year of strict adherence to GFD the quality of life of adult CD patients was higher (symptoms disappeared), but then it significantly decreased (probably due to the difficulty in strict maintenance of GFD) [29]. Mazzone et al. demonstrated that children and teenagers with CD were more likely to experience emotional and behavioral problems, particularly loneliness and depression, than their healthy peers [30]. Parents rated the quality of life of their CD children lower than the children themselves [31].

## 5. Conclusions

Over the last 10 years, an increase in the number of children with celiac disease adhering to the gluten-free diet has been observed in the West Pomeranian region. Similarly, to other countries, today every fourth child with celiac disease does not follow a gluten-free diet. 

For years, it was teenagers who most often failed to adhere to the gluten-free diet. The lack of awareness of the course of the disease, the increasing independence from parents, the need to buy food and prepare meals for themselves, the need to better organize everyday life as well as the environmental pressure are the factors resulting in more frequent failure to follow GFD. Teenagers require psychological support and repeated instruction explaining to them that they cannot eat even the smallest amounts of gluten, even though it does not cause any intestinal symptoms at this age, but unfortunately is harmful to their health.

Over the years, the frequency of non-intentional non-adherence to the gluten-free diet has significantly decreased as a result of better dietary care.

Systematic medical and dietary supervision contributes to more effective treatment of children with celiac disease, which in turn ensures their proper development and prevents complications. 

## Figures and Tables

**Table 1:** Number of children covered by the study in 2006–2007 and in 2016–2017.

Child’s Age (years)	Number of Children		Σ
2006–2007	2006–2007	2016–2017	
0–12	26	24	50
13–18	22	30	52
0–18	48	54	102
